# Supplementary material for: Stepwise modifications of transcriptional hubs link pioneer factor activity to a burst of transcription
Source: Nat Commun. 2023 Aug 10;14:4848. doi: 10.1038/s41467-023-40485-6 (PMC10415302; doi:10.1038/s41467-023-40485-6)
Supplement: Supplementary file 1 — Supplementary Information [file 41467_2023_40485_MOESM1_ESM.pdf]

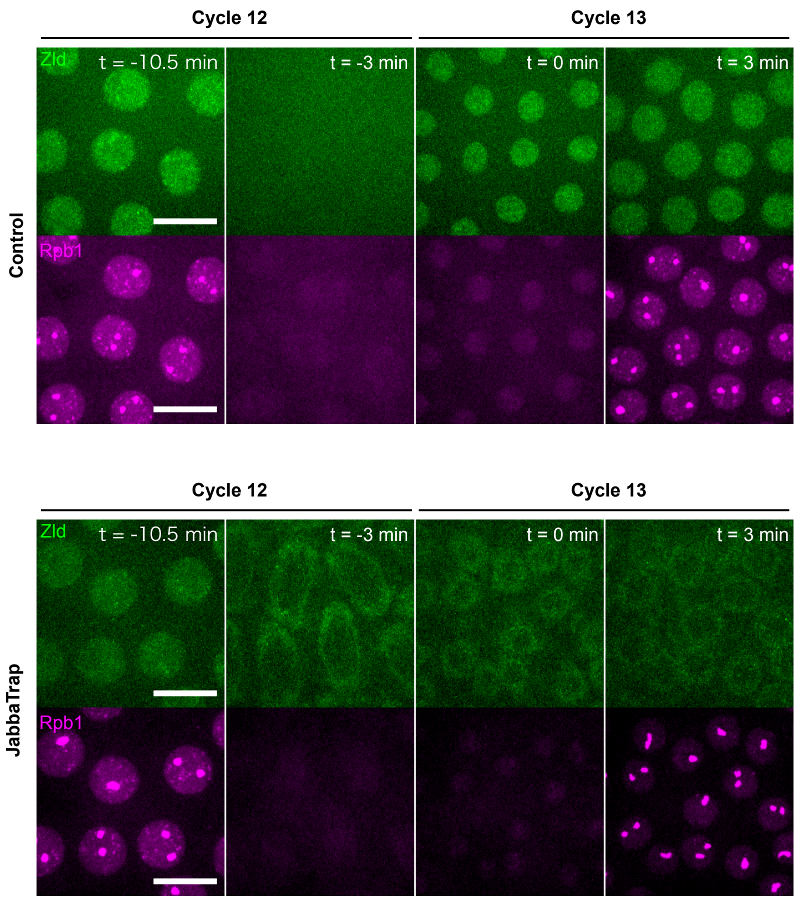

Supplementary Fig. 1. Abrupt sequestration of Zelda by JabbaTrap in mitosis 12 blocks RNAPII clustering at non-histone genes in cycle 13. Representative stills from live imaging of sfGFP-Zld and mCherry-Rpb1 in embryos injected either water (top) or JabbaTrap mRNA (bottom). Time relative to the start of cycle 13 is indicated. The JabbaTrap expressed from injected mRNA is anchored to lipid droplets in the cytoplasm and unable to effectively sequester its nuclear targets until nuclear membrane breakdown in mitosis (compare the minus-3-min frame to the control). Post-mitotic nuclear re-accumulation of Zld was blocked (0-min and 3-min frames). This abrupt inhibition of nuclear Zld function during mitosis 12 blocked RNAPII clustering except the large clusters at histone locus bodies (HLBs) in the following cycle 13 (see the 3-min frame). Maximal projections are shown. Similar outcomes were observed in 3 embryos. Scale bars, 10 μm.

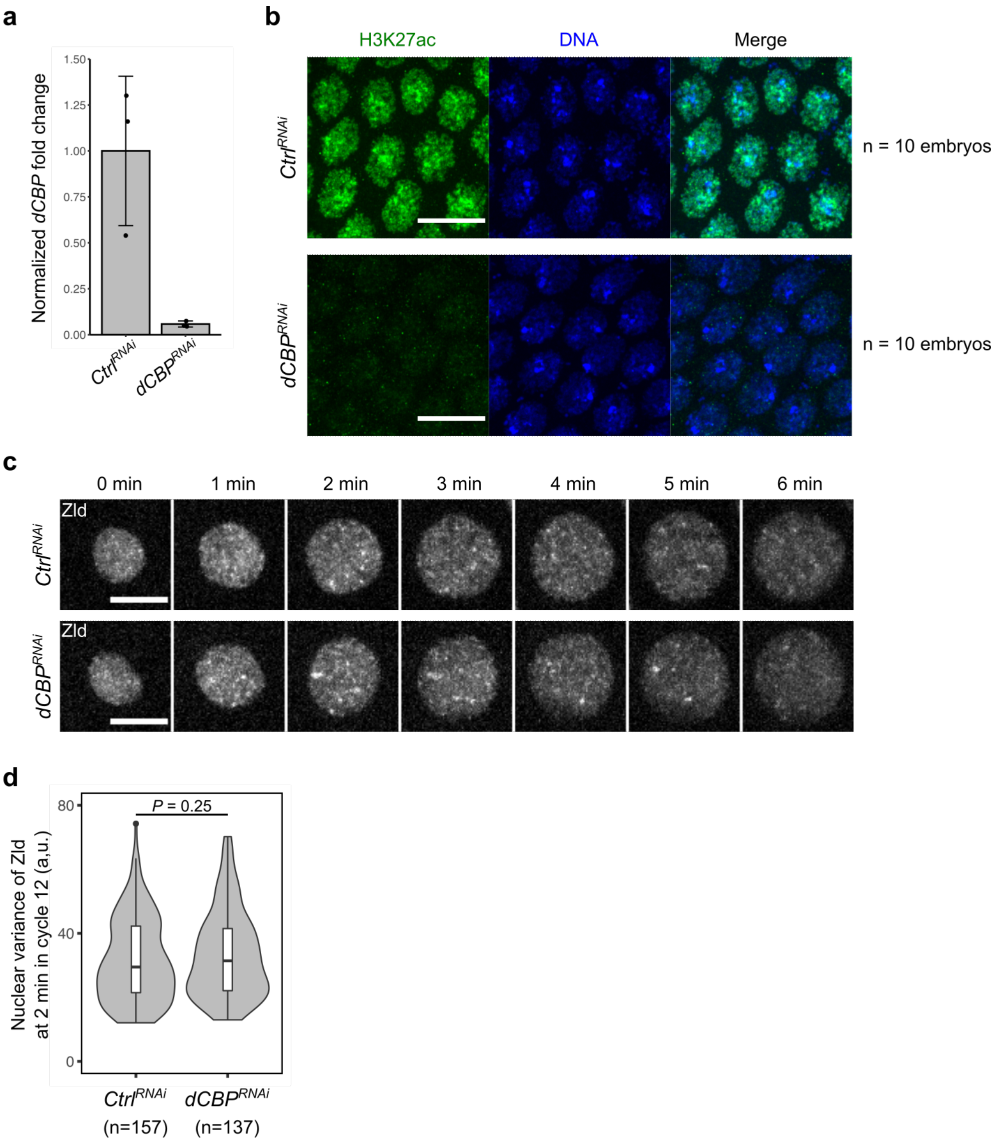

Supplementary Fig. 2. Validation and characterization of *dCBP* knockdown in early embryos. a, RT-qPCR quantification of *dCBP* transcript levels normalized by the reference gene *RpL32* in 0-1.5-hour old embryos maternally expressing shRNA targeting *mCherry* (control) or *dCBP*. Each data point represents a biological replicate. Data are presented as mean ± SD (n = 3 biological replicates). **b,** Representative images of H3K27ac immunostaining in embryos with RNAi knockdown for *mCherry* (control) or *dCBP*. Maximal projections are shown. Scale bars, 10 μm. **c,** Representative stills from live imaging of mNeonGreen-Zld in embryos expressing shRNA targeting *mCherry* (control) or *dCBP*. Time relative to the start of interphase 12 is indicated. Maximal projections are shown. The number of embryos (*n*) observed with similar outcomes is indicated. Scale bars, 5 μm. **d,** Violin plots with overlaid box plots showing the variance of mNeonGreen-Zld intensities in nuclei from embryos expressing shRNA targeting *mCherry* (control) or *dCBP* at 2 minutes in cycle 12. The number of nuclei (*n*) pooled from 6 embryos is indicated. The central lines of the box plots represent median. Whiskers extend to 1.5 times interquartile range from the box. Data points for outliers are shown. *P* value was calculated by a two-sided Mann-Whitney U test. Source data are provided as Source Data file.

a

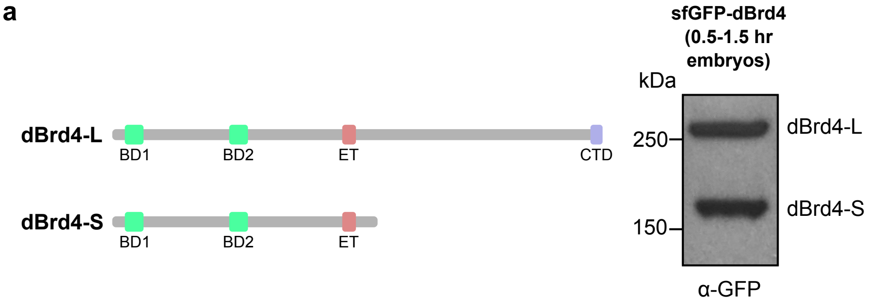

b

Genotype	% hatched
w ^{1118}	92 (n = 200)
sfGFP-dBrd4	92.5 (n = 200)
HaloTag-dBrd4	92.5 (n = 200)

c

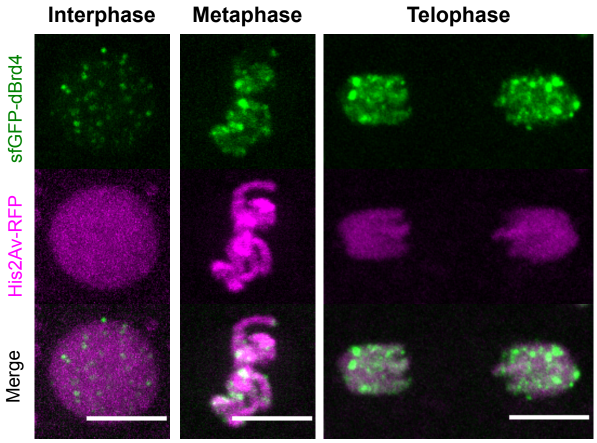

Supplementary Fig. 3. Characterization of endogenously tagged dBrd4. (**a,** left), schematic diagrams of the long and short isoforms of dBrd4. BD, bromodomain. ET, extraterminal domain. CTD, C-terminal domain. (**a**, right), western blotting of sfGFP-dBrd4 in protein lysates from 0.5-1.5 hr embryos using an anti-GFP antibody. **b,** Hatch rates of embryos from mothers of indicated genotypes. The zygotic genotypes are the same as that of mothers. Embryos were incubated for 36 hours at 25°C before scoring the number of hatched embryos. **c,** Snapshots from live imaging of sfGFP-dBrd4 and His2Av-RFP during indicated cell-cycle phases in cycle 12. Maximal projections are shown. Similar outcomes were observed in 3 embryos. Scale bars, 5 μm.

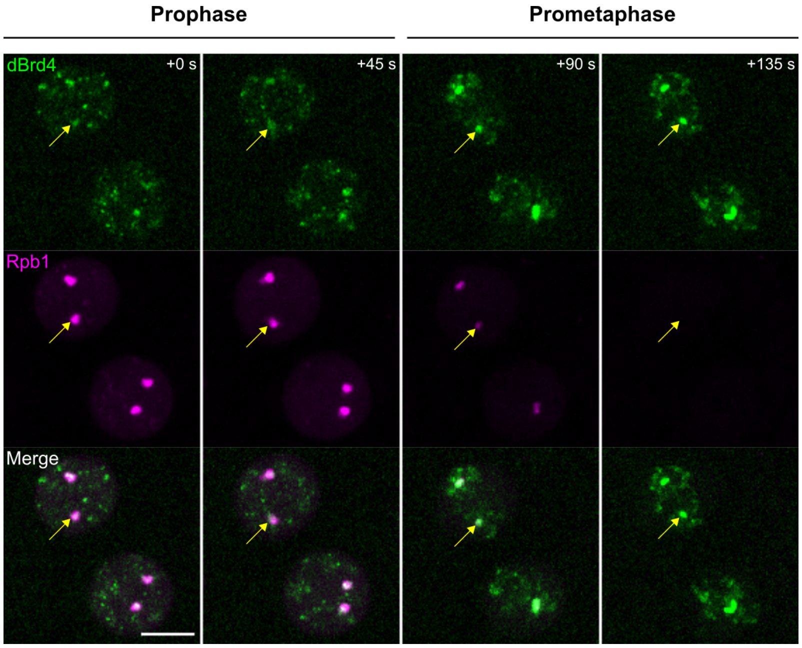

Supplementary Fig. 4. The most prominent dBrd4 clusters on mitotic chromosomes are localized at histone genes. Representative stills from live imaging of sfGFP-dBrd4 and mCherry-Rpb1 when nuclei were going into mitosis 12, here visualized as the compaction of chromosomes coated by dBrd4. Time relative to the start of the movie is indicated. Arrows point to one of the histone locus bodies, initially marked by large Rpb1 clusters and later by dBrd4. Maximal projections are shown. Similar outcomes were observed in 3 embryos. Scale bar, 5 μm.

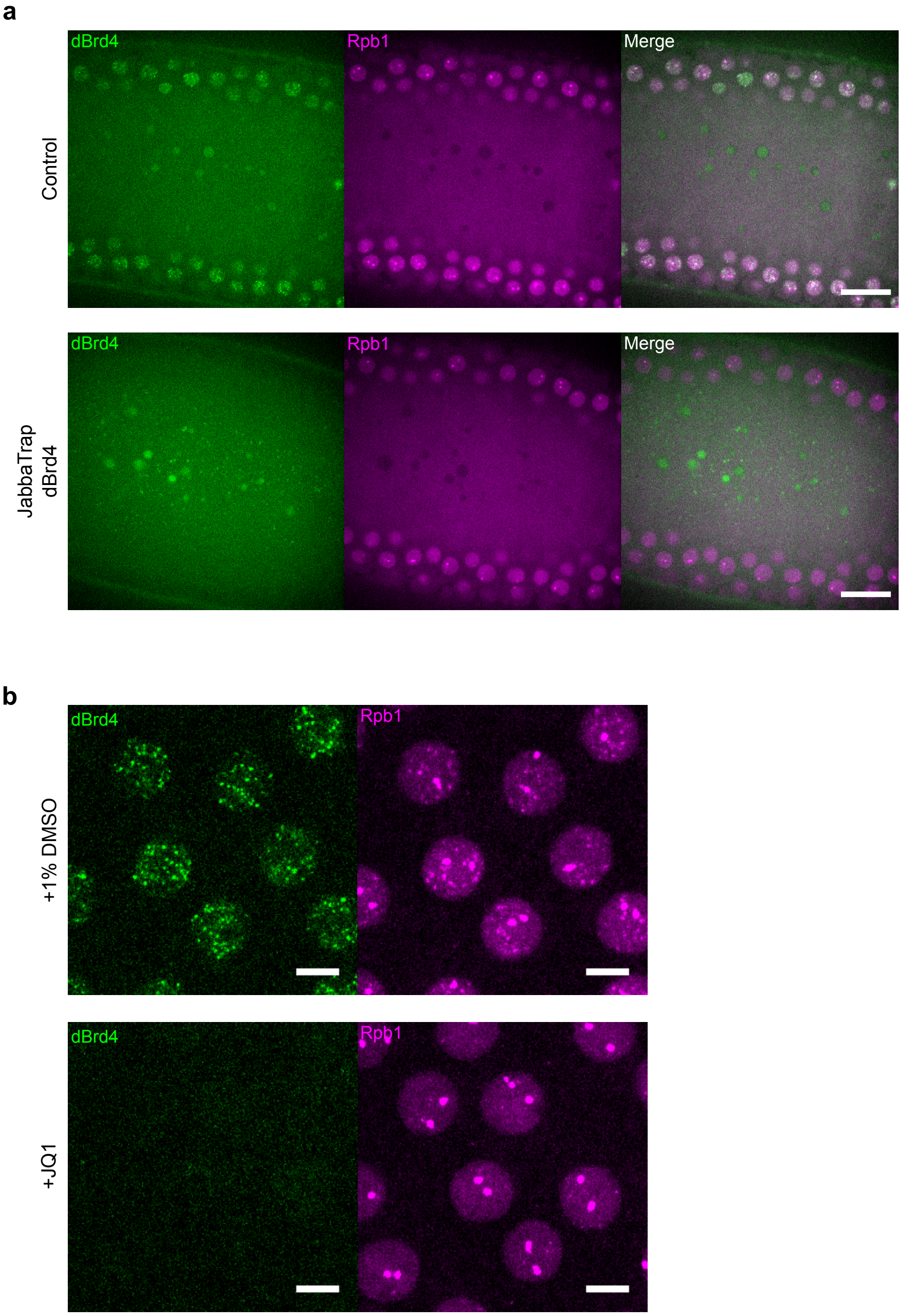

Supplementary Fig. 5. Functional inhibition of sfGFP-dBrd4 by cytoplasmic sequestration or the JQ1 inhibitor. a, Single-z-plane images from live imaging of sfGFP-dBrd4 and mCherry-Rpb1 in syncytial embryos with or without JabbaTrap. The control embryo was from the following mother: *w, sfGFP-dBrd4, mCherry-Rpb1*. The JabbaTrap embryo was from the following mother: *w, sfGFP-dBrd4, mCherry-Rpb1; ; Mat-tub-Gal4/+, UAS-JabbaTrap-bcd3'UTR/+.* The central part of the embryos along the anterior-posterior axis are shown. In the JabbaTrap embryo, sfGFP-dBrd4 was sequestered to lipid droplets visualized as puncta in the interior of the embryo. Scale bars, 10 μm. Similar outcomes were observed in 6 embryos. **b,** Snapshots from live imaging of sfGFP-dBrd4 and mCherry-Rpb1 at 3-4 minutes in cycle 12 in embryos injected with 1% DMSO as control or JQ1, a bromodomain inhibitor of Brd4. Maximal projections are shown. Similar outcomes were observed in 3 embryos. Scale bars, 5 μm.

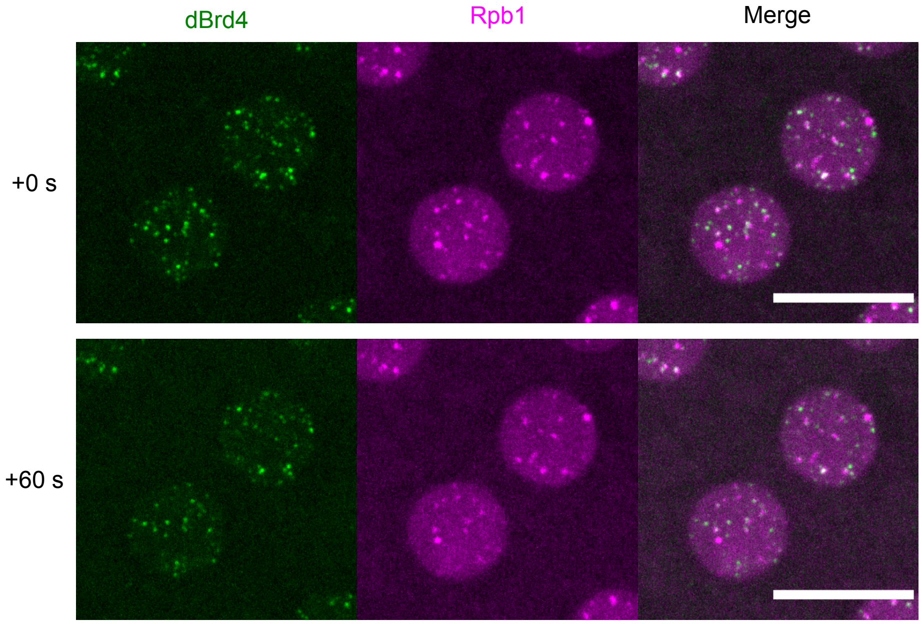

Supplementary Fig. 6. The injection of 2% formaldehyde rapidly arrests nuclear division cycle and immobilizes dBrd4 and RNAPII clusters. Two consecutive frames from time-lapse imaging of sfGFP-dBrd4 and mCherry-Rpb1 in an embryo fixed by formaldehyde injection. The injection was performed at 3 minutes after mitosis 11, and the embryo was further incubated for 8 minutes before taking the first frame. Maximal projections are shown. Similar outcomes were observed in 3 embryos. Scale bars, 10 μm.

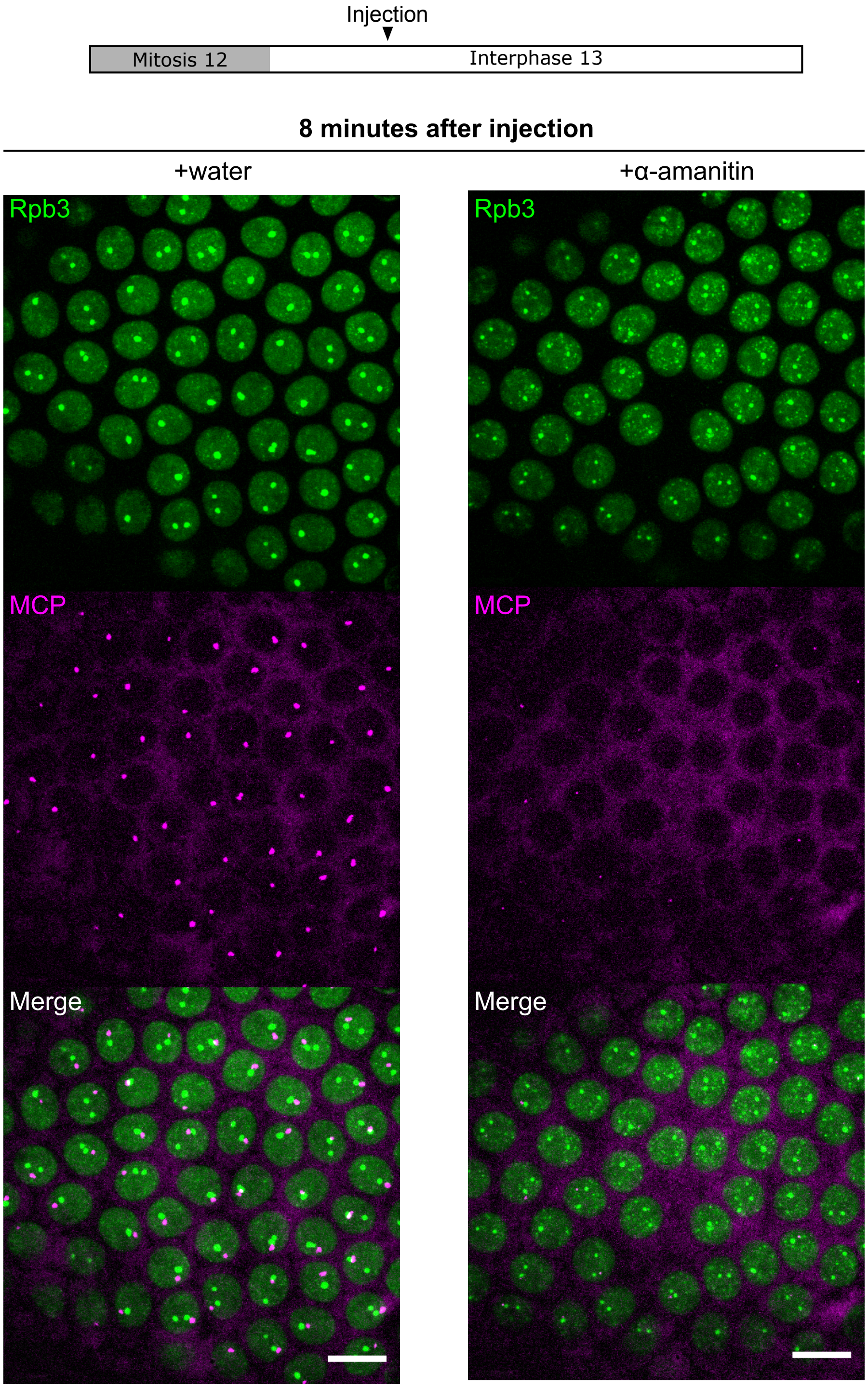

Supplementary Fig. 7. Abrupt injection of α-amanitin attenuates transcription and stabilizes small RNAPII clusters. (Top), Schematic diagram of the experimental design as described in Fig. 7a. (Bottom), Representative stills from live imaging of EGFP-Rpb3 and MCP-mCherry in embryos carrying the *hbP2-MS2* reporter and abruptly injected with water or α-amanitin around 3.5 minutes into interphase 13. Maximal projections at 8 minutes after injection are shown. The anterior (left) parts of the embryos are displayed. Similar outcomes were observed in 5 embryos for each treatment. Scale bars, 10 μm.

Supplementary Movie 1. Time-lapse imaging of sfGFP-dBrd4 and mCherry-Rpb1 during cycle 11.

**Supplementary Table 1. *Drosophila melanogaster* lines used in this study**	
**Genotype**	**Source**
nos-Cas9	Bloomington Drosophila Stock Center (#78781)
w, mCherry-Rpb1, sfGFP-Zld	This paper
y[1] sc[*] v[1] sev[21]; P{y[+t7.7] v[+t1.8]=TRiP.GL00094}attP2 (expresses dsRNA for RNAi of w under UAS control)	Bloomington Drosophila Stock Center (#35573)
y[1] sc[*] v[1] sev[21]; P{y[+t7.7] v[+t1.8]=VALIUM20-mCherry}attP2 (expresses dsRNA for RNAi of mCherry under UAS control)	Bloomington Drosophila Stock Center (#35785)
UASp-shRNA.zld (expresses dsRNA for RNAi of zld under UAS control)	Sun et al., 2015
y[1] sc[*] v[1] sev[21]; P{y[+t7.7] v[+t1.8]=TRiP.HMS01570}attP2/TM3, Sb[1] (expresses dsRNA for RNAi of nej under UAS control)	Bloomington Drosophila Stock Center (#36682)
w; EGFP-Rpb3; UASp-shRNA.w	This paper
w; EGFP-Rpb3; UASp-shRNA.zld	This paper
w; EGFP-Rpb3; UASp-shRNA.nej	This paper
w; Mat-tub-Gal4; Mat-tub-Gal4	Bloomington Drosophila Stock Center (#80361)
w; EGFP-Rpb3, Mat-tub-Gal4; Mat-tub-Gal4	This paper
mNeonGreen-Zld; Mat-tub-Gal4	This paper
w, HaloTag-dBrd4	This paper
w, sfGFP-dBrd4	This paper
w, sfGFP-dBrd4; ; His2Av-mRFP	This paper
w, HaloTag-dBrd4, mNeonGreen-Zld	This paper
w, sfGFP-dBrd4, mCherry-Rpb1	This paper
w, sfGFP-dBrd4; ; UASp-shRNA.w	This paper
w, sfGFP-dBrd4; ; UASp-shRNA.zld	This paper
w, sfGFP-dBrd4; ; UASp-shRNA.nej	This paper
w, sfGFP-dBrd4; ; Mat-tub-Gal4	This paper
w; ; UASp-JabbaTrap-bcd3'UTR	This paper
w, mCherry-Rpb1; ; UASp-JabbaTrap-bcd3'UTR	This paper
w,mCherry-Rpb1; ; Mat-tub-Gal4	This paper
w, sfGFP-dBrd4, mCherry-Rpb1; ; UASp-JabbaTrap-bcd3'UTR	This paper
w, sfGFP-dBrd4, mCherry-Rpb1; Mat-tub-Gal4	This paper
nos-MCP-mCherry	Personal gift from Michael Stadler (postdoc in the laboratory of Michael Eisen)
w; EGFP-Rpb3; nos-MCP-mCherry	Cho et al., 2022
y[1] w[*]; P{hbP2-MS2-lacZ}JB38F	Bloomington Drosophila Stock Center (#60338)

Supplementary Table 2. Females used for embryo collection and experiments in this study

**Figure**	**Label**	**Genotype**
**Main text**		
1a	Control; JabbaTrap Zelda	*w, mCherry-Rpb1, sfGFP-Zld*
1b; 1c		*w, mCherry-Rpb1, sfGFP-Zld*
1d	Ctrl^{RNAi}; Zld^{RNAi}; dCBP^{RNAi}	*w; EGFP-Rpb3/EGFP-Rpb3, Mat-tub-Gal4/+; Mat-tub-Gal4/+, UASp-shRNA/+*
2a; 2b	Ctrl^{RNAi}; Zld^{RNAi}; dCBP^{RNAi}	*w, sfGFP-dBrd4; ; Mat-tub-Gal4/+, UASp-shRNA/+*
2c; 2d	JabbaTrap control	*w, mCherry-Rpb1; Mat-tub-Gal4/+, UASp-JabbaTrap-bcd3'UTR/+*
2c; 2d	JabbaTrap dBrd4	*w, sfGFP-dBrd4, mCherry-Rpb1; Mat-tub-Gal4/+, UASp-JabbaTrap-bcd3'UTR/+*
3		*w, HaloTag-dBrd4/+, mNeonGreen-Zld/mNeonGreen-Zld*
4		*w, sfGFP-dBrd4, mCherry-Rpb1*
5a		*w, sfGFP-dBrd4, mCherry-Rpb1*
5b		*w, mCherry-Rpb1; EGFP-Rpb3*
6b		*mNeonGreen-Zld*
6d		*w, sfGFP-dBrd4, mCherry-Rpb1*
6f		*w, sfGFP-dBrd4, mCherry-Rpb1*
7b; 7d		*w, sfGFP-dBrd4, mCherry-Rpb1*
7f; 7g		*w; EGFP-Rpb3; MCP-mCherry* (crossed to hbP2-MS2 males)
**Supplementary Figures**		
1	Control; JabbaTrap	*w, mCherry-Rpb1, sfGFP-Zld*
2a; 2b	Ctrl^{RNAi}; dCBP^{RNAi}	*w; EGFP-Rpb3/EGFP-Rpb3, Mat-tub-Gal4/+; Mat-tub-Gal4/+, UASp-shRNA/+*
2c; 2d	Ctrl^{RNAi}; dCBP^{RNAi}	*mNeon-Zld/+; Mat-tub-Gal4/+; UASp-shRNA/+*
3c		*w, sfGFP-dBrd4; ; His2Av-mRFP*
4		*w, sfGFP-dBrd4, mCherry-Rpb1*
5a	Control	*w, mCherry-Rpb1; Mat-tub-Gal4/+, UASp-JabbaTrap-bcd3'UTR/+*
5a	JabbaTrap dBrd4	*w, sfGFP-dBrd4, mCherry-Rpb1; Mat-tub-Gal4/+, UASp-JabbaTrap-bcd3'UTR/+*
5b		*w, sfGFP-dBrd4, mCherry-Rpb1*
6		*w, sfGFP-dBrd4, mCherry-Rpb1*
7		*w; EGFP-Rpb3; MCP-mCherry* (crossed to *hbP2-MS2* males)
