# Supplementary material for: Stepwise modifications of transcriptional hubs link pioneer factor activity to a burst of transcription
Source: Nat Commun. 2023 Aug 10;14:4848. doi: 10.1038/s41467-023-40485-6 (PMC10415302; doi:10.1038/s41467-023-40485-6)
Supplement: Supplementary file 3 — Description of Additional Supplementary Files [file 41467_2023_40485_MOESM3_ESM.pdf]

Description of Additional Supplementary Files

File Name: Supplementary Movie 1
Description: Time-lapse imaging of sfGFP-dBrd4 and mCherry-Rpb1 during cycle 11.
