# Supplementary material for: Stepwise modifications of transcriptional hubs link pioneer factor activity to a burst of transcription
Source: Nat Commun. 2023 Aug 10;14:4848. doi: 10.1038/s41467-023-40485-6 (PMC10415302; doi:10.1038/s41467-023-40485-6)
Supplement: Supplementary file 5 — Reporting Summary [file 41467_2023_40485_MOESM5_ESM.pdf]

Corresponding author(s): Patrick O'Farrell
Last updated by author(s): Jul 18, 2023

Reporting Summary

Nature Portfolio wishes to improve the reproducibility of the work that we publish. This form provides structure for consistency and transparency in reporting. For further information on Nature Portfolio policies, see our Editorial Policies and the Editorial Policy Checklist.

Statistics

For all statistical analyses, confirm that the following items are present in the figure legend, table legend, main text, or Methods section.

n/a	Confirmed	
☐	☒	The exact sample size (*n*) for each experimental group/condition, given as a discrete number and unit of measurement
☒	☐	A statement on whether measurements were taken from distinct samples or whether the same sample was measured repeatedly
☐	☒	The statistical test(s) used AND whether they are one- or two-sided<br>*Only common tests should be described solely by name; describe more complex techniques in the Methods section.*
☒	☐	A description of all covariates tested
☒	☐	A description of any assumptions or corrections, such as tests of normality and adjustment for multiple comparisons
☐	☒	A full description of the statistical parameters including central tendency (e.g. means) or other basic estimates (e.g. regression coefficient) AND variation (e.g. standard deviation) or associated estimates of uncertainty (e.g. confidence intervals)
☐	☒	For null hypothesis testing, the test statistic (e.g. *F*, *t*, *r*) with confidence intervals, effect sizes, degrees of freedom and *P* value noted<br>*Give P values as exact values whenever suitable.*
☒	☐	For Bayesian analysis, information on the choice of priors and Markov chain Monte Carlo settings
☒	☐	For hierarchical and complex designs, identification of the appropriate level for tests and full reporting of outcomes
☐	☒	Estimates of effect sizes (e.g. Cohen's *d*, Pearson's *r*), indicating how they were calculated

Our web collection on statistics for biologists contains articles on many of the points above.

Software and code

Policy information about availability of computer code

Data collection	Data were acquired using Volocity 6 software (Quorum Technologies).
Data analysis	Image processing was performed in FIJI/ImageJ (version 2.9.0). Tracking and quantification of MCP foci were performed using TrackMate (version 7.10.2) in FIJI. All other quantifications were performed in Python 3 using standard and open-sourced libraries, including NumPy and scikit-image. Graphs were plotted using ggplot2 in R (version 3.2.1).

For manuscripts utilizing custom algorithms or software that are central to the research but not yet described in published literature, software must be made available to editors and reviewers. We strongly encourage code deposition in a community repository (e.g. GitHub). See the Nature Portfolio guidelines for submitting code & software for further information.

Data

Policy information about availability of data

All manuscripts must include a data availability statement. This statement should provide the following information, where applicable:

- Accession codes, unique identifiers, or web links for publicly available datasets
- A description of any restrictions on data availability
- For clinical datasets or third party data, please ensure that the statement adheres to our policy

All data generated in this study have been deposited at the Zenodo database (DOI: 10.5281/zenodo.8136965). Any additional information is available from the corresponding author upon request.

Human research participants

Policy information about studies involving human research participants and Sex and Gender in Research.

Reporting on sex and gender	N/A
Population characteristics	N/A
Recruitment	N/A
Ethics oversight	N/A

Note that full information on the approval of the study protocol must also be provided in the manuscript.

Field-specific reporting

Please select the one below that is the best fit for your research. If you are not sure, read the appropriate sections before making your selection.

☒ Life sciences ☐ Behavioural & social sciences ☐ Ecological, evolutionary & environmental sciences

For a reference copy of the document with all sections, see nature.com/documents/nr-reporting-summary-flat.pdf

Life sciences study design

All studies must disclose on these points even when the disclosure is negative.

Sample size	No statistical method was used to determine sample size.<br>The sample size of three biological replicates for RT-qPCR was chosen based on standard practice in the field.<br>The sample size of at least 3 embryos for microscopy was chosen. We typically imaged at lease 100 nuclei in total, which is sufficient for statistical analysis. Previous studies using similar sample sizes include DOI: 10.1101/gad.321646.118
Data exclusions	No data exclusions were made.
Replication	All experimental findings were verified in at least 3 embryos, and about 30 neighboring nuclei were examined in each embryo with similar outcomes. All attempts at replication were successful.
Randomization	Not relevant. Embryos with categorized genotypes were used for experiments.
Blinding	Blinding was not possible because the same person performed sample preparation, data acquisition, and analysis.

Reporting for specific materials, systems and methods

We require information from authors about some types of materials, experimental systems and methods used in many studies. Here, indicate whether each material, system or method listed is relevant to your study. If you are not sure if a list item applies to your research, read the appropriate section before selecting a response.

Materials & experimental systems

n/a	Involved in the study	
☐	☒	Antibodies
☒	☐	Eukaryotic cell lines
☒	☐	Palaeontology and archaeology
☐	☒	Animals and other organisms
☒	☐	Clinical data
☒	☐	Dual use research of concern

Methods

n/a	Involved in the study	
☒	☐	ChIP-seq
☒	☐	Flow cytometry
☒	☐	MRI-based neuroimaging

Antibodies

Antibodies used

Rabbit anti-H3K27ac (Active Motif, #39133)
Goat anti-rabbit Alexa Fluor 488 (Thermo Fisher, #A11008)
Rabbit anti-GFP (Abcam, #ab290)
Goat anti-rabbit HRP (Thermo Fisher, #A16096)

Validation

Rabbit anti-H3K27ac is validated by the manufacturer (https://www.activemotif.com/catalog/details/39133/histone-h3-acetyl-lys27-antibody-pab). It has been used in previous studies on Drosophila embryos, such as DOI: 10.1186/s13059-016-1057-2.

Anti-rabbit Alexa Fluor 488 is validated by the manufacturer (https://www.thermofisher.com/antibody/product/Goat-anti-Rabbit-IgG-H-L-Cross-Adsorbed-Secondary-Antibody-Polyclonal/A-11008). It has been widely used for immunostaining including in Drosophila embryos, such as DOI: 10.1016/j.isci.2022.105837.

Rabbit anti-GFP (Abcam, #ab290) is validated by the manufacturer (https://www.abcam.com/products/primary-antibodies/gfp-antibody-ab290.html). It has been used for Drosophila studies, such as DOI: 10.1371/journal.pone.0083903.

Goat anti-rabbit HRP (Thermo Fisher, #A16096) is validated by the manufacturer (https://www.thermofisher.com/antibody/product/Goat-anti-Rabbit-IgG-H-L-Secondary-Antibody-Polyclonal/A16096). Previous studies using this antibody include DOI: 10.1038/s41596-022-00713-7.

Animals and other research organisms

Policy information about studies involving animals; ARRIVE guidelines recommended for reporting animal research, and Sex and Gender in Research

Laboratory animals

The following lines of Drosophila melanogaster were used in this study.
nos-Cas9
w, mCherry-Rpb1, sfGFP-Zld
y[1] sc[*] v[1] sev[21]; P{y[+t7.7] v[+t1.8]=TRiP.GL00094}attP2 (expresses dsRNA for RNAi of w under UAS control)
y[1] sc[*] v[1] sev[21]; P{y[+t7.7] v[+t1.8]=VALIUM20-mCherry}attP2 (expresses dsRNA for RNAi of mCherry under UAS control)
UASp-shRNA.zld (expresses dsRNA for RNAi of zld under UAS control)
y[1] sc[*] v[1] sev[21]; P{y[+t7.7] v[+t1.8]=TRiP.HMS01570}attP2/TM3, Sb[1] (expresses dsRNA for RNAi of nej under UAS control)
w; EGFP-Rpb3; UASp-shRNA.w
w; EGFP-Rpb3; UASp-shRNA.zld
w; EGFP-Rpb3; UASp-shRNA.nej
w; Mat-tub-Gal4; Mat-tub-Gal4
w; EGFP-Rpb3, Mat-tub-Gal4; Mat-tub-Gal4
mNeonGreen-Zld; Mat-tub-Gal4
w, HaloTag-dBrd4
w, sfGFP-dBrd4
w, sfGFP-dBrd4; ; His2Av-mRFP
w, HaloTag-dBrd4, mNeonGreen-Zld
w, sfGFP-dBrd4, mCherry-Rpb1
w, sfGFP-dBrd4; ; UASp-shRNA.w
w, sfGFP-dBrd4; ; UASp-shRNA.zld
w, sfGFP-dBrd4; ; UASp-shRNA.nej
w, sfGFP-dBrd4; ; Mat-tub-Gal4
w; ; UASp-JabbaTrap-bcd3'UTR
w, mCherry-Rpb1; ; UASp-JabbaTrap-bcd3'UTR
w,mCherry-Rpb1; ; Mat-tub-Gal4
w, sfGFP-dBrd4, mCherry-Rpb1; ; UASp-JabbaTrap-bcd3'UTR
w, sfGFP-dBrd4, mCherry-Rpb1; Mat-tub-Gal4
nos-MCP-mCherry
w; EGFP-Rpb3; nos-MCP-mCherry
y[1] w[*]; P{hbP2-MS2-lacZ}JB38F

Adult females within two weeks of eclosion were used for embryo collections. Embryos during nuclear cycle 11-14 (about 1.5-4 hours after egg laying) were imaged by confocal microscopy.

Wild animals	This study did not involve wild animals.
Reporting on sex	Sex was not considered in this study, since the early Drosophila embryogenesis is driven by the maternal program.
Field-collected samples	This study did not involve samples collected from the field.
Ethics oversight	No ethical approval or guidance was required for the study of Drosophila.

Note that full information on the approval of the study protocol must also be provided in the manuscript.
